# THE IMPACT OF AN EDUCATION INTERVENTION: IMPROVING COMMUNICATION BETWEEN OLDER ADULTS AND HEALTH CARE PROVIDERS

**DOI:** 10.1093/geroni/igz038.3316

**Published:** 2019-11-08

**Authors:** Janna Heyman, Linda White-Ryan, Peggy Kelly

**Affiliations:** 1 Fordham University, New York, United States; 2 Fordham University, New York, New York, United States; 3 Fordham University, West Harrison, New York, United States

## Abstract

As people age, ensuing physical and psychological problems can increase, which makes it paramount to be comfortable discussing medical needs with health care professionals, particularly in light of the danger associated with misunderstanding medication use and combining alcohol with prescriptions and/or over the counter medications (National Institute on Aging, 2018). National studies found that about 40 percent of adults ages 65 and older drink alcohol and often do not understand the dangers of combining alcohol with medications (National Institute for Alcohol and Alcohol Abuse, 2008). An educational intervention was developed with a team of expert physicians, nurses, pharmacists and social workers who work in gerontology to focus on improving communication and addressing alcohol and medication use for older adults. A randomized controlled trial was conducted to assess whether the educational intervention improved older adults’ comfort in communicating with their health care providers, as well as their knowledge of the concomitant use of alcohol and prescription and over-the-counter (OTC) medications. Results of a MANCOVA showed that those in the intervention group showed larger increases in scores on communication with their health providers and knowledge about the implications of combining alcohol with prescription drugs than those in the control group (Wilks’ Lamda=.808, F(3,76)=6.039, p=.001<.05). In addition, linear regression models showed that the intervention was significantly associated with participants’ knowledge of the implications of combining alcohol with prescription drugs. The coefficient across models was approximately 1.00, which represented a substantial increase given the average score of 6.5.

